# Modeling of entropy optimization for hybrid nanofluid MHD flow through a porous annulus involving variation of Bejan number

**DOI:** 10.1038/s41598-020-69458-1

**Published:** 2020-07-30

**Authors:** Zahir Shah, M. Sheikholeslami, Poom Kumam, Ikramullah, Ahmad Shafee

**Affiliations:** 1 Science Laboratory Building, Faculty of Science, Center of Excellence in Theoretical and Computational Science (TaCS-CoE), King Mongkut’s University of Technology Thonburi (KMUTT), 126 Pracha-Uthit Road, Bang Mod, Thrung Khru, Bangkok, 10140 Thailand; 2 Department of Mechanical Engineering, Babol Noshirvani University of Technology, Babol, Islamic Republic of Iran; 3 Renewable Energy Systems and Nanofluid Applications in Heat Transfer Laboratory, Babol Noshirvani University of Technology, Babol, Islamic Republic of Iran; 4 KMUTT-Fixed Point Research Laboratory, Room SCL 802 Fixed Point Laboratory, Science Laboratory Building, Department of Mathematics, Faculty of Science, King Mongkut’s University of Technology Thonburi (KMUTT), 126 Pracha-Uthit Road, Bang Mod, Thrung Khru, Bangkok, 10140 Thailand; 5 Department of Medical Research, China Medical University Hospital, China Medical University, Taichung, Taiwan; 6 Department of Physics, Kohat University of Science and Technology, Kohat, Khyber Pakhtunkhwa 26000 Pakistan; 7 Division of Computational Physics, Institute for Computational Science, Ton Duc Thang University, Ho Chi Minh City, Vietnam; 8 Faculty of Electrical and Electronics Engineering, Ton Duc Thang University, Ho Chi Minh City, Vietnam

**Keywords:** Energy science and technology, Mathematics and computing, Physics

## Abstract

We numerically investigate the non-Darcy magnetohydrodynamic hybrid nanoparticle migration through a permeable tank using control volume finite element method through entropy generation. The roles of various amounts of Permeability, Lorentz and Rayleigh (Ra) number are investigated upon the various aspects of the hybrid nanofluid flow through contour and 3-D plots. Through curve fitting technique, analytical expressions for Nu_ave_ and Bejan number as functions of Ra, Ha and Da are obtained. It is found that the strength of the vortexes decline and temperature of the inner wall augments with the higher magnetic field, while temperature drops with increasing buoyancy forces and medium permeability. The irreversibility terms associated with the generation of the thermal energy and applied magnetic field (S_gen,th_, S_gen,M_) enhance while the other terms (S_gen,f_, S_gen,p_) drop with the rising values of the magnetic field strength. These quantities show exactly opposite behavior with augmenting Da. The Bejan number drops while Nu_ave_ augments with the rising buoyancy forces. The agreement with the previous published results confirms the accuracy of the employed computational model.

## Introduction

The heat transfer analysis during fluid flow is a topic of immense importance and interest due to its industrial and technological perspectives^1–4^. The heat energy transfer through convection is the dominant mode which carries heat energy during a fluid flow. The free convection flow is a general and widespread phenomenon, which occurs in various industrial and scientific domains^5,6^. The usages of Lorentz drastically change the fluid flow pattern. The Lorentz force arises retards the flow, hence decreases the convection. The fluid flow is mainly controlled by the strength and direction of the applied forces. The MHD flow has numerous scientific and technological applications, like in crystal growth, nuclear reactors, electronic and solar systems etc. The different aspects of MHD convection flow are investigated both experimentally and theoretically by various researchers^7–9^.

In the recent past nanofluids have been introduced with goal of augmenting the heat transfer capability of the conventional fluids. Generally, nanofluids are produced when nanoparticles of metal (metal oxides) are mixed uniformly throughout the base liquids. The size and geometry of the nanoparticles have an influential role in augmenting the thermal conductivities of ordinary liquids. The basic concepts of nanoliquids and its various applications can be found in the references^10–13^. An analytical study on nanofluid about heat transfer in normal convection flow in the wavy cone was carried out by Iqbal and Mehmood^14^. The results were shown that Titanium Oxide and Cooper as nanopowders had maximum heating and cooling performance respectively. Moreover, the results in this study have been an important data reference for tracing the enactment of natural convection heat transfer inside the wavy cone. Zhou and Jiang^15^ studied heating properties of nanomaterial surface traction driven by convection within an enclosure. As a result, nanofluid ${\text{Al}}_{2} {\text{O}}_{3}$—distilled water indicated a nonmonotonic change for thermal transfer performance and convective intensity with the increase of nanoparticles volume concentration while thermal transfer performance and convective intensity of nanofluid decreased monotonously for ZnO–PGW nanofluid. In addition, the entropy generation and flow properties with various volume concentrations of nanoparticles are completely analyzed. Sheikholeslami et al.^16^ scrutinized how ambient magnetic field can alter the thermal properties of a nanomaterial moving through a porous container confronting an elliptical shape obstacle. Shah et al.^17^ examined the Hall impact and thermal radiations impacts on the flow of Titania nanoluid mixed with various pure fluids on a tilted rotating wall. Mebarek Oudina^18^ examined the convective migration of Titania nanofluid through a cylindrical container by considering a discrete source of heat energy. Shah et al.^19–21^ studied the various characteristics associated with the nanofluid flow by including the electric, magnetic and Hall effects.

Currently, there is a growing tendency in investigating the heat carrying capabilities of hybrid nanomaterial^22–25^. In such type of fluid more than one type of nanomaterial is mixed with the base liquid. Hybrid nanofluids exhibit various thermo-physicals and chemical attributes which do not possess by a single component. Hybrid nanoliquids can be divided into different kinds^26–28^. A lot of undertaken numerical and experimental research work confirms that hybrid nanofluid is more proper than simple one. Suresh et al.^29^ utilized the copper-alumina nanoparticles with employing two-step approach. Suresh et al.^30^ also scrutinized the advantages of hybrid nano powder (copper–alumina) for thermal system. They involved water as pure fluid.

We know that the heat energy transfer process is also accompanied by entropy generation due to the phenomenon of thermodynamic irreversibility. The existence of temperature gradients, dissipation, and the characteristics of heat transportation due to convective mode are the main causes which can generate entropy. A direct relation exists between energy dissipation and irreversibility during a given process. The thermodynamics 2nd law indicates that, the entropy optimization rate in a given process (thermodynamically) must be entropy positive. We can regulate the efficiency of a system by minimizing its entropy optimization rate. The entropy determines the direction and magnitude of changes that occur. Bejan^31^ scrutinized the idea of irreversibility optimization in a convective heat energy transfer process. Polidori and his friend^32^ simulated thermal transfer of Newtonian nanofluids in laminar natural convection. They observed that the model of viscosity has an important part in performance and temperature is not specified only by nanomaterial conductivity. The phenomenon of two significant slip mechanism of Jeffery fluid consisting of nano-liquid is studied by Rehman et al.^33^ through entropy generation. Ellahi et al.^34^ investigated the effects produced due to different shapes of the suspended nanoparticles by employing entropy generation technique. Miroshnichenko and colleagues^35^ considered a steady magnetic in a trapezoidal cavity as a fractional fully open with the presence of CuO nanofluid to study of the normal convection. The result was revealed that Nu declines by the growth of Hartmann number, while it improves by the increase of nanoparticles volume concentration. Zubair et al.^36^ performed the Darcy–Forchheimer nanomaterial flow of different Nanomaterials by using entropy optimization. The recent research work about entropy optimization with various interesting effects can be read in^37–39^.

In the current work, we want to numerically simulate the hybrid nanofluid (MWCNT and Fe_3_O_4_ mixed with water) flow through a permeable enclosure involved to uniform magnetic force with the help of entropy optimization. In “Explanation of geometry” section, we sketch the schematics of the problem. The formulation and numerical solution is given in third part. The entropy analysis was presented in fourth part. The discussion about the best mesh size and validation of the obtained results through the FORTRAN code are explained in “Grid independence and code validation” section. The numerical results are discussed through different contour and 3-D plots in “Results and discussion” section. Conclusion of current modeling study was summarized in part 7.

## Explanation of geometry

**Figure 1:**
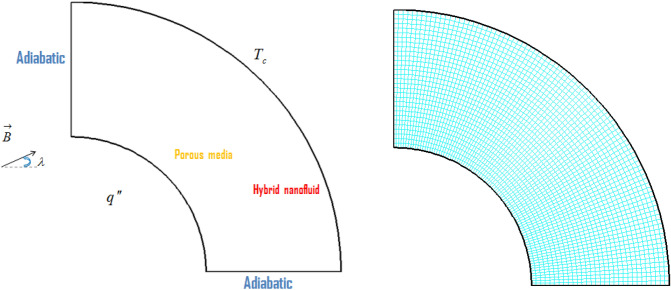
Permeable enclosure filled with hybrid nanomaterial.

The geometrical description of the present investigation is explained in Fig. 1. Two of the container walls are adiabatic, while the outer surface is maintained cooled at temperature T_c_, and the inner surface is maintained hot due to a uniform heat power. The container is filled with hybrid nanomaterials (MWCNT and Fe_3_O_4_) dispersed in the testing liquid (water). Non-Darcy approach is employed in order to simulate the dynamics of the nanomaterial in the permeable cavity. Horizontal **B** is employed to control the migration of nanomaterial. To get the solution of final equations with higher accuracy, we utilize the computational method developed by Sheikholeslami^16^, namely the control volume finite element method (CVFEM).

## Problem formulation and numerical solution

In this section we first model the problem through appropriate mathematical equations and then discuss its numerical solution. We consider the 2-dimensional hybrid nanomaterial flow in a permeable medium in the presence of a heat source. A uniform **B** is employed in the x–y plane. The dynamical equations governing the two-dimensional hybrid nanomaterial flow in the existence of Lorentz force are as under:

1 $$\left( {\frac{{\partial^{2} v}}{{\partial x^{2} }} + \frac{{\partial^{2} v}}{{\partial y^{2} }}} \right)\mu_{nf} + B_{x} \sigma_{nf} B_{y} u - \frac{{\mu_{nf} }}{K}v - \frac{\partial P}{{\partial y}} + g\left( {T - T_{c} } \right)\rho_{nf} \beta_{nf} - B_{x} vB_{x} \sigma_{nf} = \left( {\frac{\partial v}{{\partial x}}u + v\frac{\partial v}{{\partial y}}} \right)\rho_{nf} ,$$

2 $$\left( {v\frac{\partial T}{{\partial y}} + u\frac{\partial T}{{\partial x}}} \right)\left( {\rho C_{p} } \right)_{nf} = \left( {\frac{{\partial^{2} T}}{{\partial y^{2} }} + \frac{{\partial^{2} T}}{{\partial x^{2} }}} \right)k_{nf} ,$$

3 $$\begin{aligned} & \left( {\rho_{nf} } \right)\left( {\frac{\partial u}{{\partial y}}v + u\frac{\partial u}{{\partial x}}} \right) = \sigma_{nf} B_{x} B_{y} v - \sigma_{nf} B_{y}^{2} u + \left( {\frac{{\partial^{2} u}}{{\partial y^{2} }} + \frac{{\partial^{2} u}}{{\partial x^{2} }}} \right)\mu_{nf} - \frac{\partial P}{{\partial x}} - \frac{{\mu_{nf} }}{K}u, \\ & \left( {B_{x} ,B_{y} } \right) = \left( {\cos \lambda ,\sin \lambda } \right) \\ \end{aligned}$$

4 $$\frac{\partial v}{{\partial y}} + \frac{\partial u}{{\partial x}} = 0.$$

here T, P, $\rho$, and ${C}_{p}$ are the temperature, pressure, density and heat capacity, respectively. $\sigma_{nf}$ is nanomaterial electrical conductivity. In order to eliminate the pressure components, vorticity formulations are considered as:

5 $$\begin{aligned} & \frac{\partial u}{{\partial y}} - \frac{\partial v}{{\partial x}} = - \omega , \\ & - \frac{\partial \psi }{{\partial x}} = v,\frac{\partial \psi }{{\partial y}} = u \\ \end{aligned}$$

To get the non-dimensional forms, the following transformations are employed:

6 $$U = \frac{uL}{{\alpha_{f} }},\quad V = \frac{vL}{{\alpha_{f} }},\quad \Theta = \left[ {q^{{\prime \prime }} L/k_{f} } \right]^{ - 1} \left( {T - T_{c} } \right),\left( {X,Y} \right) = \frac{{\left( {x,y} \right)}}{L}.$$

here “*k*_*f*_” is fluid thermal conductivity. Using these transformations, we get the above equations [Eqs. (5) and (6)] in transformed forms as:

7 $$\left( {\frac{{\partial^{2} \Theta }}{{\partial X^{2} }} + \frac{{\partial^{2} \Theta }}{{\partial Y^{2} }}} \right) = \frac{\partial \Theta }{{\partial Y}}V + U\frac{\partial \Theta }{{\partial X}},$$

8 $$\, - \Omega = \frac{{\partial^{2} \Psi }}{{\partial X^{2} }} + \frac{{\partial^{2} \Psi }}{{\partial Y^{2} }},$$

9 $$\begin{aligned} & \Pr \left( {\frac{{A_{3} A_{2}^{2} }}{{A_{1} A_{4}^{2} }}} \right)Ra\left( {\frac{\partial \Theta }{{\partial X}}} \right) + \,\Pr A_{6} A_{2} Ha^{2} \left( {\left( {\cos \gamma } \right)\frac{\partial U}{{\partial X}}\left( {\sin \lambda } \right) - \left( {0.5\sin 2\lambda } \right)\frac{\partial V}{{\partial Y}}} \right)\left[ {\frac{1}{{A_{4} A_{1} }}} \right] \\ & \quad + \,\Pr \left( {\frac{{A_{5} A_{2} }}{{A_{1} A_{4} }}} \right)\left( {\frac{{\partial^{2} \Omega }}{{\partial Y^{2} }} + \frac{{\partial^{2} \Omega }}{{\partial X^{2} }}} \right) + \,\Pr \left[ {\frac{{A_{6} A_{2} }}{{A_{1} A_{4} }}} \right]\left( { - \frac{\partial V}{{\partial X}}\left( {\cos \lambda } \right)^{2} + \left( {\sin \lambda } \right)^{2} \frac{\partial U}{{\partial Y}}} \right)Ha^{2} \\ & \quad - \,\frac{\Pr }{{Da}}\Omega \left( {\frac{{A_{5} A_{2} }}{{A_{1} A_{4} }}} \right) = \frac{\partial \Omega }{{\partial Y}}V + U\frac{\partial \Omega }{{\partial X}}. \\ \end{aligned}$$

where the different A’s and other symbols designate the following quantities:

10 $$\begin{aligned} & A_{6} = \frac{{\sigma_{nf} }}{{\sigma_{f} }},\quad A_{2} = \frac{{\left( {\rho C_{P} } \right)_{nf} }}{{\left( {\rho C_{P} } \right)_{f} }},\quad A_{1} = \frac{{\rho_{nf} }}{{\rho_{f} }},\quad Ra = \beta_{f} g\,L^{4} q^{{\prime \prime }} {/}\left( {\alpha_{f} \upsilon_{f} k_{f} } \right), \\ & \quad A_{3} = \frac{{\left( {\rho \beta } \right)_{nf} }}{{\left( {\rho \beta } \right)_{f} }},\quad A_{4} = \frac{{k_{nf} }}{{k_{f} }},\quad Ha = L\left( {\mu_{f} {/}\sigma_{f} } \right)^{ - 0.5} B_{0} , \\ & \quad Da = \frac{K}{{L^{2} }},\quad A_{5} = \frac{{\mu_{nf} }}{{\mu_{f} }},\quad \Pr = \upsilon_{f} {/}\alpha_{f} . \\ \end{aligned}$$

## Entropy analysis

We are modeling the MHD hybrid nanofluid non Darcy flow through a permeable enclosure with the help of entropy optimization. Irreversibility or entropy generation is generally associated with the heat transfer phenomena. From 2nd law, it is established that the entropy of an irreversible process is always positive. There are different sources for entropy generation: for example, the existence of concentration gradient, temperature gradient, viscous dissipation, convective heat energy transformation characteristics etc. The best thermal system can be designed through the second law of thermodynamics by minimizing the thermodynamic irreversibility.

For analyzing the first and second laws behavior of the non Darcy MHD hybrid nanofluid flow through entropy optimization, the following important parameters are defined:

11 $$Be = \left( {S_{gen,total} } \right)^{ - 1} S_{gen,th}$$

12 $$X_{d} = T_{0} S_{gen,total}$$

13 $$\begin{aligned} S_{gen,total} &= \underbrace {{\left( {v^{2} + u^{2} } \right)\frac{{\mu_{nf} }}{TK}}}_{{S_{gen,P} }} + \underbrace {{\mu_{nf} \left[ {2\left( {\left( {\frac{\partial v}{{\partial y}}} \right)^{2} + \left( {\frac{\partial u}{{\partial x}}} \right)^{2} } \right) + \left( {\frac{\partial u}{{\partial y}} + \frac{\partial v}{{\partial x}}} \right)^{2} } \right]\frac{1}{{T^{2} }}}}_{{S_{gen,f} }} \\ & \quad + \underbrace {{T^{ - 2} \left[ {\left( {T_{x} } \right)^{2} + \left( {T_{y} } \right)^{2} } \right]k_{nf} }}_{{S_{gen,th} }} + \underbrace {{\frac{{\sigma_{nf} }}{{T^{2} }}B_{0}^{2} v^{2} }}_{{S_{gen,M} }} \\ \end{aligned}$$

14 $$Nu_{ave} = \frac{1}{S}\int\limits_{0}^{s} {A_{4} \Theta^{ - 1} } \,ds$$

here Be in Eq. (11) is the Bejan number, that is the ratio of entropy generated due to thermal energy flow with the total generated entropy. The symbol X_d_ on the L.H.S of Eq. (12) is the exergy loss during the flow. In Eq. (13), S_gen,total_ is the total entropy generated during the MHD hybrid nanofluid flow through a permeable annulus which is the sum of different irreversibility terms arise due to various contributions. The first term (S_gen,p_) on the R.H.S of Eq. (13) is the irreversibility due to the permeability of the medium. The fourth and second terms (S_gen,M_, S_gen,f_) are the entropies associated with the applied MHD and the frictional forces due to the fluid motion. The third term (S_gen,th_) arises due to the heat energy flow as a result of temperature gradient exists inside the fluid. Finally, the symbols Nu_ave_ in Eq. (14) is the average Nusselt number.

## Grid independence and code validation

**Figure 2:**
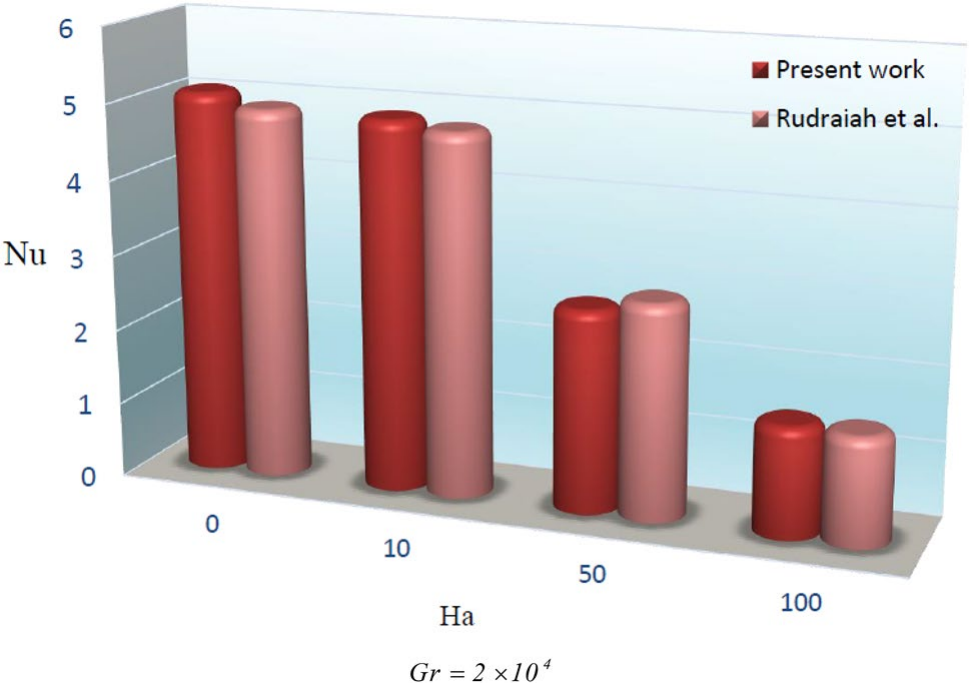
Comparison of the present results obtained through FORTRAN code with the former data^40^.

For a reliable numerical simulation, its output must be independent of the mesh size. Table 1 shows that for $Ra = 10^{5}$, $Ha = 1,Da = 100$ and $\phi = 0.04$ the outputs for different mesh sizes are almost equal, and therefore it can be employed to simulate the flow. The results during this investigation through numerical simulation via the CVFEM and the published work^40^ are compared in Fig. 2. The small difference between the two results confirms the accuracy of our employed numerical technique CVFEM.

**Table 1:** Analysis of grid independence.

*Ra* = 105, *Ha* = 1, *Da* = 100 and *ϕ* = 0.04					
_Grid_	$61 \times 181$	$71 \times 211$	$81 \times 241$	$91 \times 271$	$101 \times 301$
Nu_ave_	5.9127	5.9165	5.9220	5.9243	5.9277

## Results and discussion

In current part of context, we scrutinize the influence of changing of Hartmann (Ha), Rayleigh (Ra) and Darcy (Da) numbers over the different aspects of non-Darcy MHD hybrid nanofluid flow through a porous enclosure by using entropy production.

**Figure 3:**
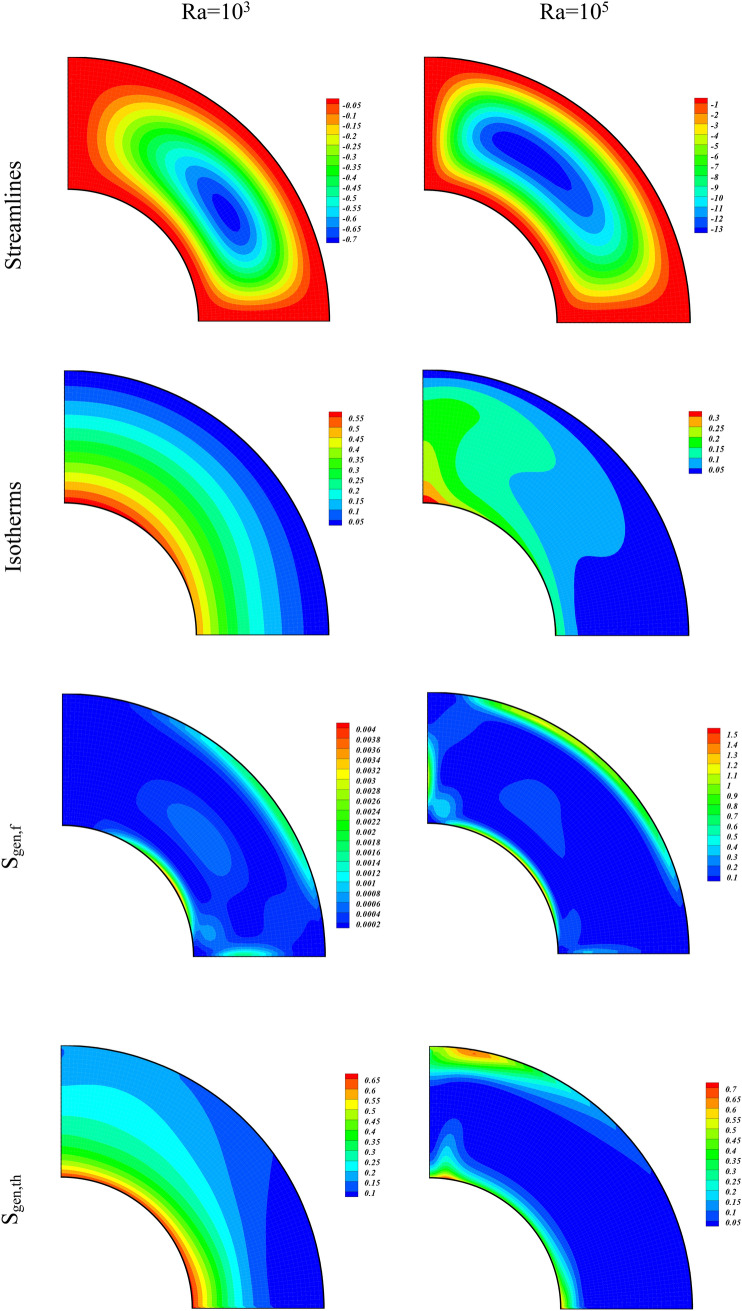

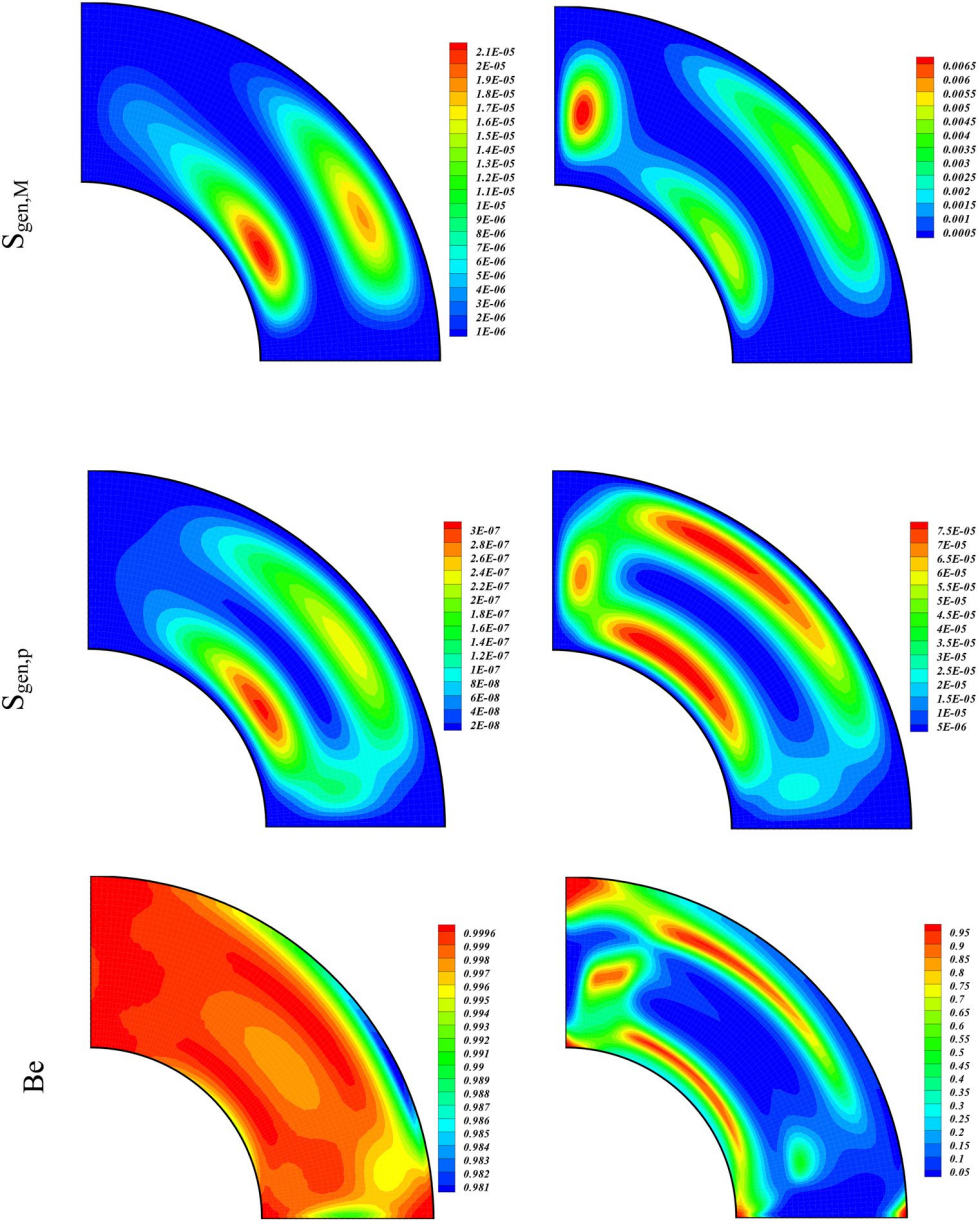
Contour plots of streamlines, isotherms, entropy terms (S_gen,f_, S_gen,th_, S_gen,M_, S_gen,p_), and Be for varying values of Ra at $\phi = 0.04,Ha = 1,Da = 100.$

The role of the buoyancy forces on the Streamlines, Isotherms, Irreversibility effects due to entropy generation (S_gen,f_, S_gen,th_, S_gen,M_, S_gen,p_), and Be for various amounts of Ra (10^3^, 10^5^) are displayed through contour plots in Fig. 3. The amounts of the other variables were taken as $\phi =0.04, Ha=1, Da=100$. The contours on the left column of Fig. 3 are plotted for Ra = 10^3^ while on the right column are plotted for Ra = 10^5^. The contours of the first panel are for the Streamlines of the hybrid nanofluid flow. We observe that at higher Ra, the strength of the streamlines contours augments and it confine to smaller region as compared to the streamlines contours at smaller Ra. This shows that the higher buoyancy forces due to the larger values of Ra constricts the hybrid nanofluid flow. The contours for the Isotherms for different Ra are drawn in the second panel of Fig. 3. It is observed from these contours, that the temperature of the inner wall drops with the augmentation of the buoyancy forces. Thus for larger Ra, the hybrid nanofluid flow carries away heat energy at higher rate from the hotter wall which causes a reduction in its temperature. The entropic terms (S_gen,f_, S_gen,th_, S_gen,M_, S_gen,p_) are respectively drawn for varying values of Ra from 3rd to 6th panel of Fig. 3. The irreversibility term S_gen,f_, which is directly related with the fluid velocity gradient augments with the higher values of Ra. We see an opposite behavior for S_gen,th_ which is associated with the lower gradient in temperature. The enhancing Ra augments both S_gen,M_ and S_gen,p_ as can be seen from the contour plots for these two entropy generated terms. This shows that the increasing buoyancy forces augment the system irreversibility due to MHD and medium permeability terms S_gen,M_ and S_gen,p_, respectively. The Bejan number (Be) drops with the higher value of Ra which is due to the thermal irreversibility term S_gen,th_, which also drops with higher Ra.

**Figure 4:**
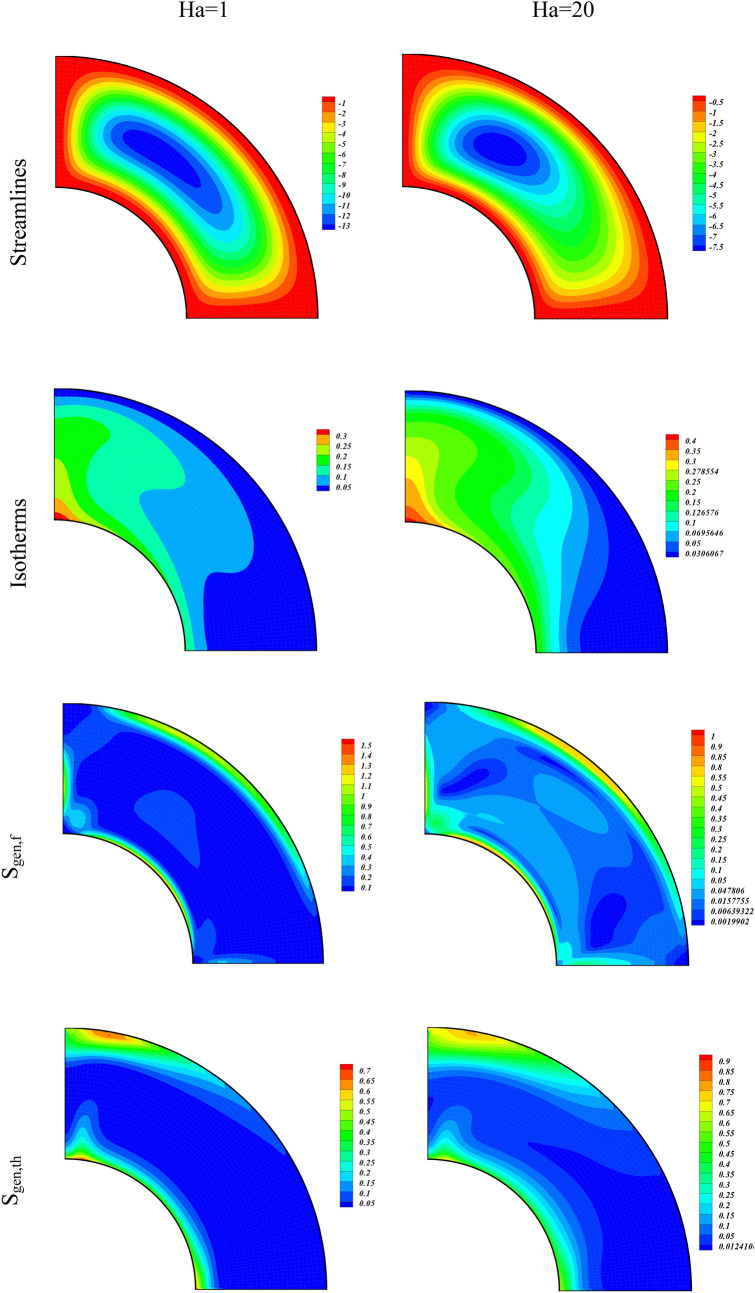

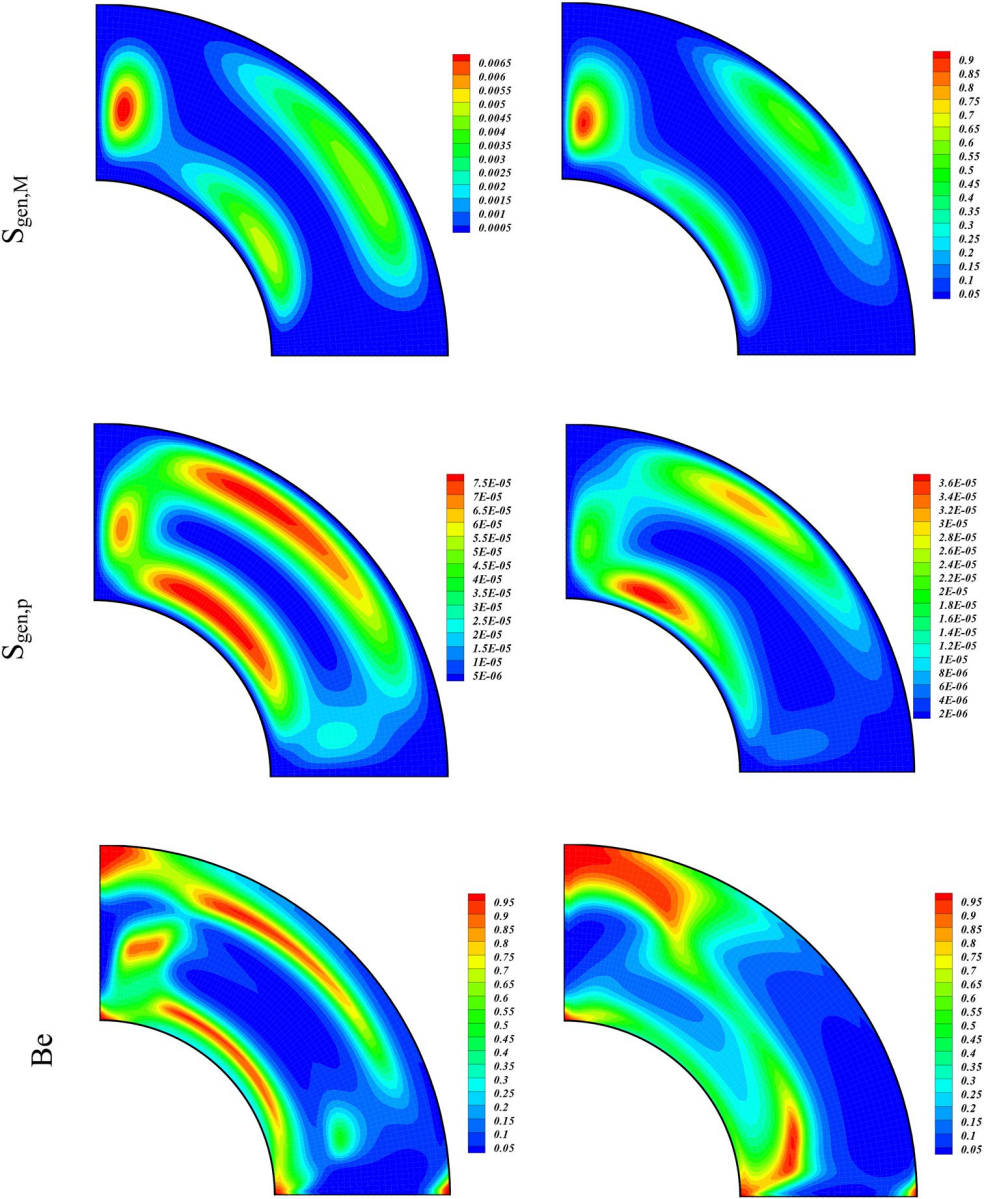
Contour plots of streamlines, isotherms, entropy terms (S_gen,f_, S_gen,th_, S_gen,M_, S_gen,p_), and Be for varying values of Ha at $\phi = 0.04,Ra = 10^{5} ,Da = 100.$

We have analyzed the simulation results for varying magnetic field (with rising Ha) through contour plots of Streamlines, Isotherms, Irreversibility effects (S_gen,f_, S_gen,th_, S_gen,M_, S_gen,p_), and Bejan number in Fig. 4. The values of the other parameters used are $\phi =0.04, Ra= {10}^{5}, Da=100$. The contour plots of the left column of Fig. 4 are plotted for Ha = 01, while that of the right column are plotted for Ha = 20. The contours of the first panel are for the Streamlines of the hybrid nanofluid with augmenting Ha. We observe an overall decline in the strength of the vortexes with the increasing Ha. This reduction in the power of vortexes shows that the larger magnetic forces decrease the hybrid nanofluid flow. We also see a rise in the temperature of the inner wall due to increasing Ha, as an output of the larger Lorentz force that increases the rate of nanoparticles collisions. The irreversibility terms (S_gen,f_, S_gen,p_) drop, whereas the entropic terms ^(^S_gen,th_, S_gen,M_) augment with the increasing electromagnetic forces (higher value of Ha). This shows that the larger magnetic forces causes to augment the irreversibility associated with the thermal energy and applied magnetic field while reduce the S_gen_ due to fluid friction and permeability of the medium. The Bejan number also enhances due to larger electromagnetic force as a result of higher Hartmann number. The increment in the Bejan number shows that S_gen,th_ participates a dominant character in the S_gen_ as compared to the contributions from the other entropy generated terms.

**Figure 5:**
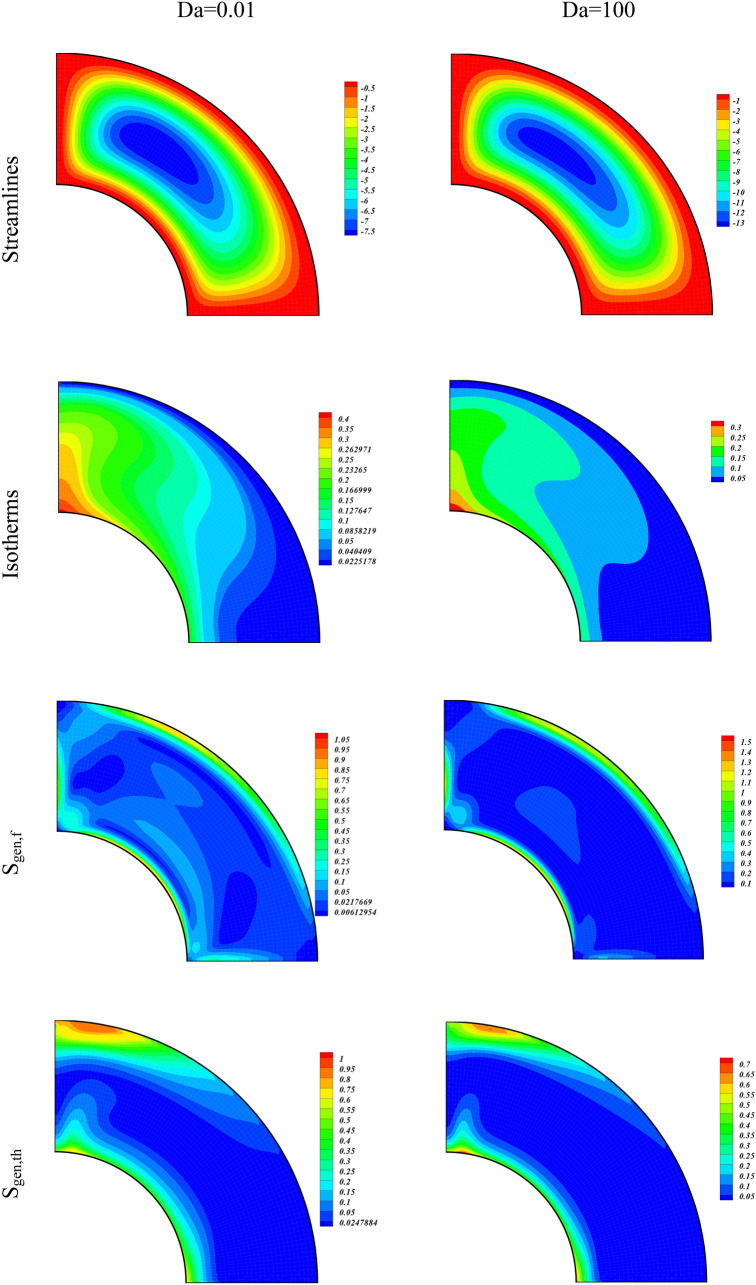

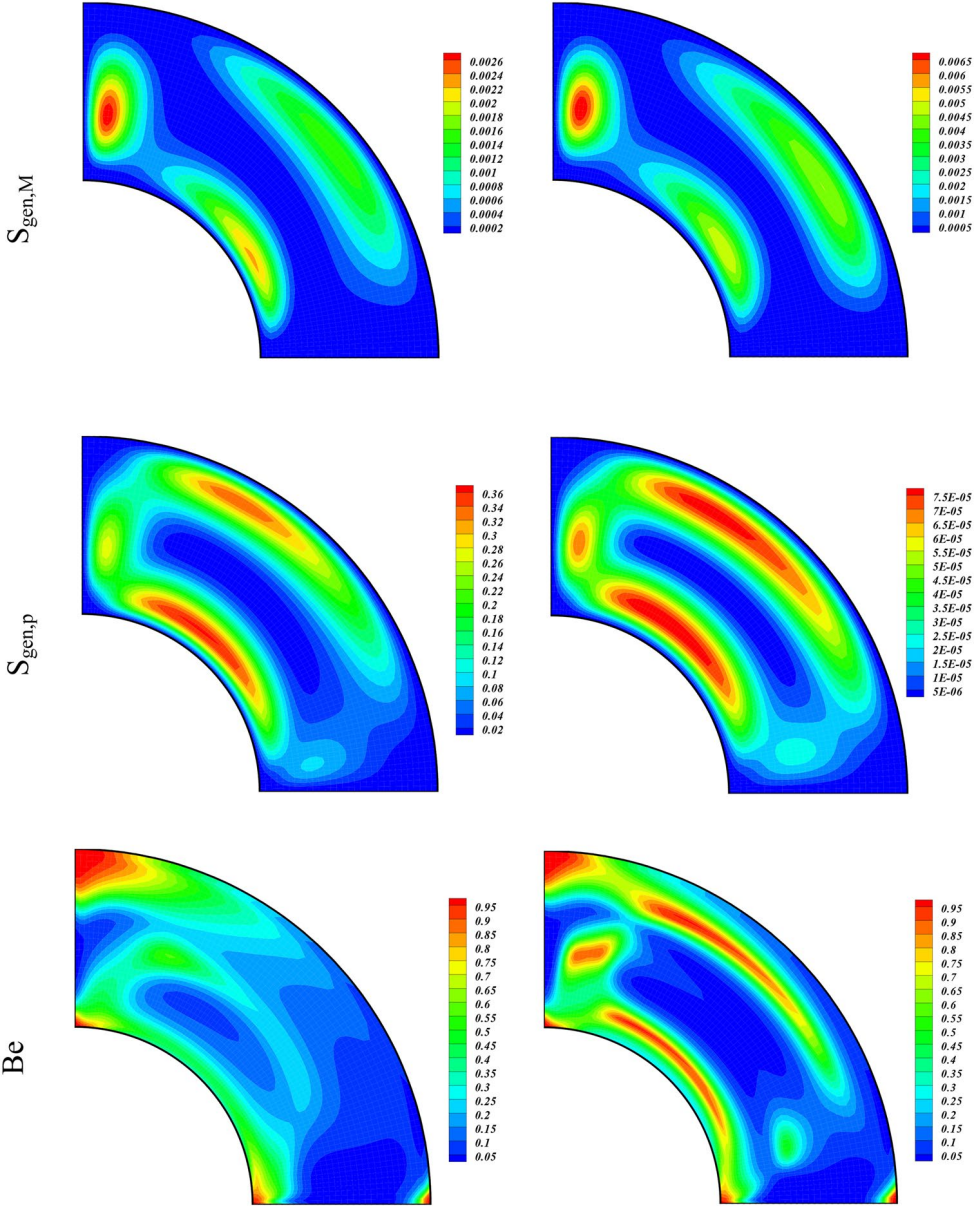
Contour plots of streamlines, isotherms, entropy terms (S_gen,f_, S_gen,th_, S_gen,M_, S_gen,p_), and Be for varying values of Da at $\phi = 0.04,Ra = 10^{5} ,Ha = 1.$

The simulation results due to the variation of the permeability of the porous medium (Da = 0.01, 100) over the related quantities of interests are displayed through contour plots in Fig. 5 by using $\phi =0.04, Ra= {10}^{5}, Ha=01$. The strength of the eddies augment and isotherms drop with the higher amount of the permeability. The entropy generated terms (S_gen,f_, S_gen,m_) augment, whereas (S_gen,th_, S_gen,p_) drop with the higher value of the Darcy number. We also observe a minute increase in the strength of the contour plot of the Bejan number with the increasing Da.

We have obtained the following two formulas for the manipulation of Be and Nu_ave_ as functions of Ha, Da, and Ra from the simulation profiles through curve fitting:

15 $$\begin{aligned} Nu_{ave} &= 3.41 - 0.29Ha - 0.07Ha\,Da + 1.47\log \left( {Ra} \right) \\ & \quad + \,0.18Da - 0.28Ha\log \left( {Ra} \right) + 0.16Da\,\log \left( {Ra} \right) \\ \end{aligned}$$

16 $$\begin{aligned} Be &= 0.64 + 0.011Ha - 5.3 \times 10^{ - 3} Da\,\log \left( {Ra} \right) - 0.36\log \left( {Ra} \right) \\ & \quad + \,4.89 \times 10^{ - 3} Da + 0.01\log \left( {Ra} \right)Ha - 7.96 \times 10^{ - 3} Ha\,Da \\ \end{aligned}$$

**Figure 6:**
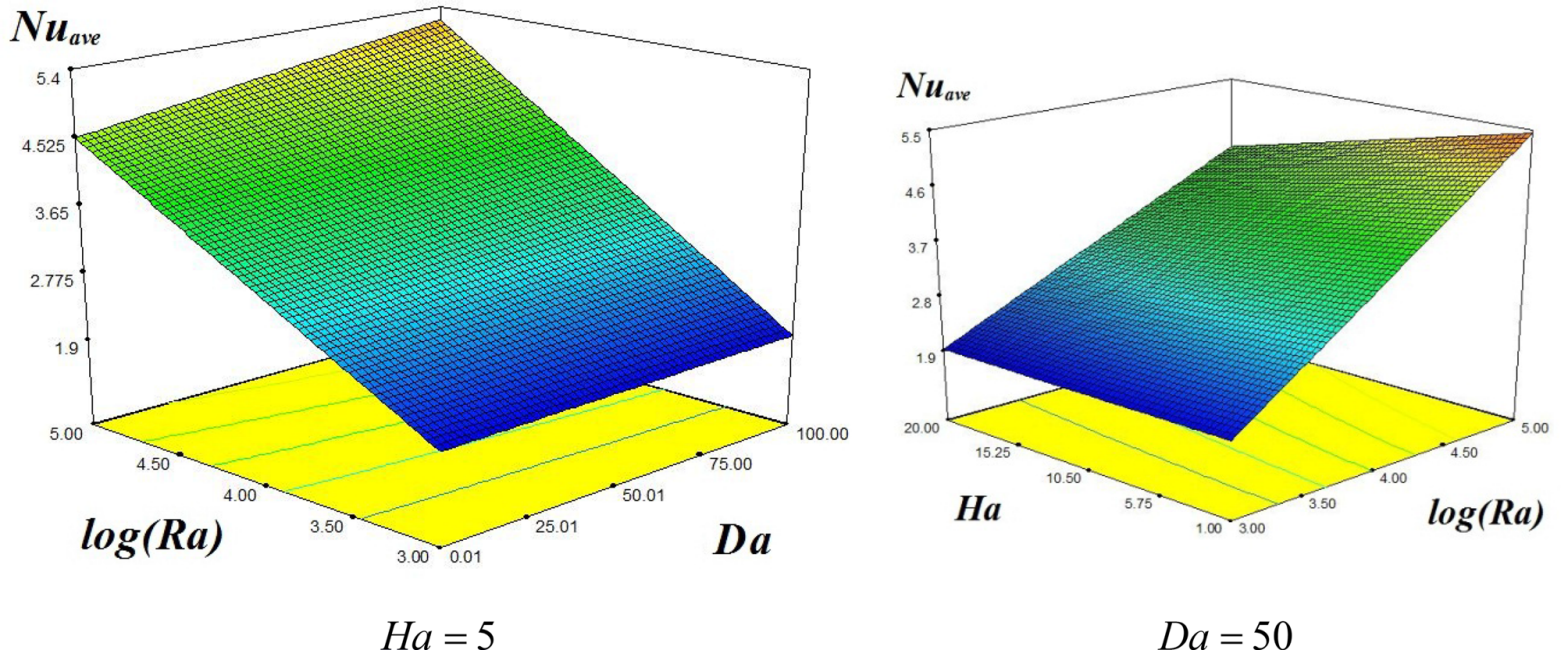
3-D plots showing the values of Nu_ave_ with (left) varying Ra and Da at Ha = 5 and $\phi = 0.04$ (right) varying values of Ha and Ra at Da = 50 and $\phi = 0.04$.

Figure 6 is the 3-D plots for the Nu_ave_ for various amounts of Ra, Da, and Ha. The left plot of Fig. 6 is the graphical display of Nu_ave_ as function of log (Ra) and Da at fixed Ha, while the right plot depicts Nu_ave_ as function of Ha and log (Ra) at fixed Da, respectively. For the left plot we have taken $\phi =0.04, Ha=5$, while for the right plot we used $\phi =0.04, Da=50$. It is clear from the left plot that Nu_ave_ augment with the higher values of Rayleigh number as well as Darcy number at fixed Ha. The rate of increase of Nu_ave_ with respect to augmenting Ra is much larger as compared to the enhancing Da. The enhancement in Nu_ave_ with respect to rising values of Da takes place at larger Ra values as cleared from the figure. From the right plot it is observed that Nu_ave_ augments with higher values of Da while drops with the augmenting Ha at fixed Ra. The rate of decrease of Nu_ave_ with higher Ha is more effective at larger values of Da.

**Figure 7:**
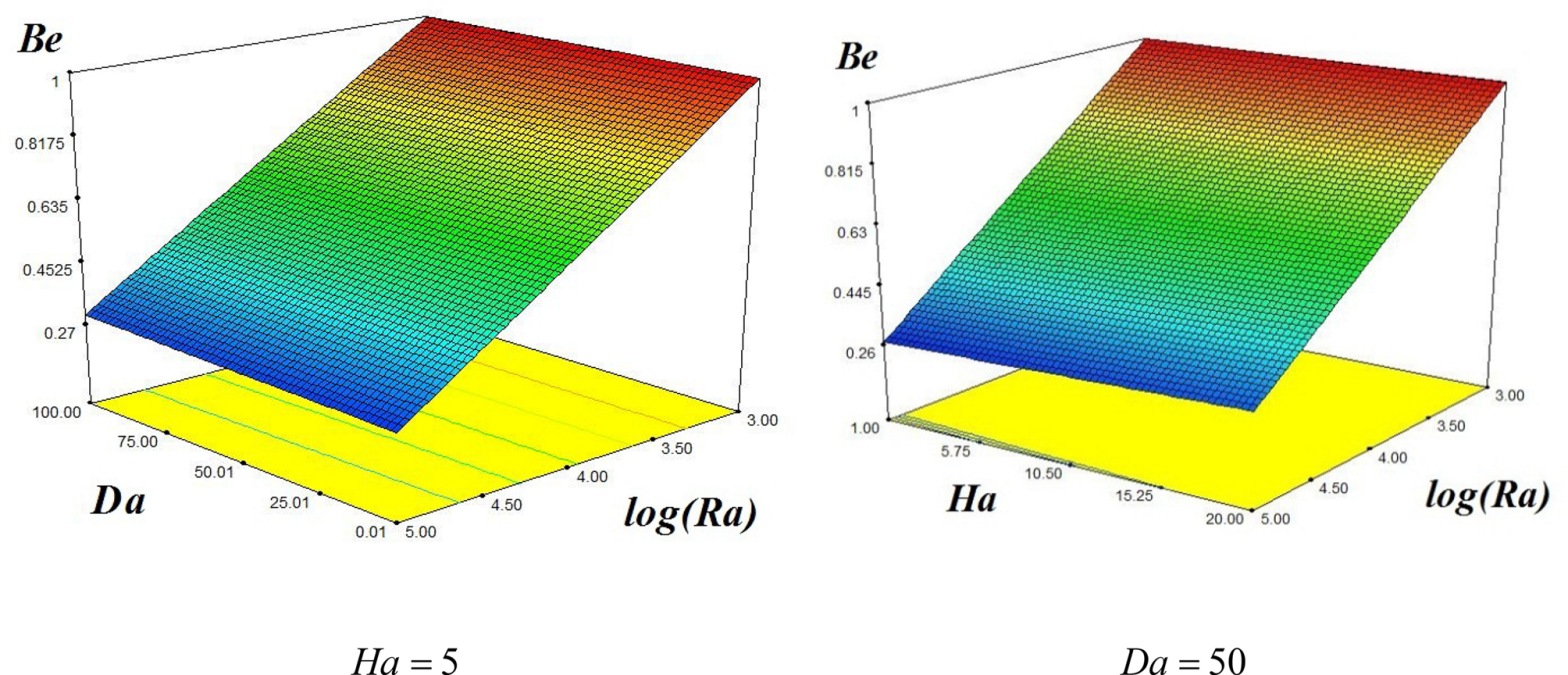
3-D plots showing the amounts of Be with (left) varying values of Ra and Da at Ha = 5 and $\phi = 0.04$ (right) varying values of Ha and Ra at Da = 50 and $\phi = 0.04$.

Figure 7 is the 3-D plots for the behavior of Bejan number (Be) with imposing different amounts of $Ha, Da, and Ra$. The left plot of Fig. 7 depicts the variation of Be with respect to varying values of Da and log (Ra) at fixed Ha, while the right plot displays Nu_ave_ as function of Ha and log (Ra) at fixed Da. For the left plot we have taken $\phi =0.04, Ha=5$, while for the right plot we used $\phi =0.04, Da=50$, respectively. We see form these plots that Be varies inversely with the higher values of Ra, while remains constant with the higher values of Da and Ha.

## Conclusions

In this section we conclude our research findings. We numerically investigated the hybrid non-Darcy nanofluid flow through entropy generation by employing the computational technique of CVFEM. The effects produced by varying amounts of Lorentz, Ra and permeability on the non-Darcy MHD hybrid nanofluid flow are investigated through contour plots of Streamlines, Isotherms, entropy generated due to different contributions and Bejan number. The relations for Be and Nu_ave_ regarding changes of Ra, Ha and Da are also obtained through curve fitting. We observe that.

1. The strength of the vortexes decline and temperature of the inner wall augment with the higher values of Ha.
2. The entropy generated terms S_gen,f_ and S_gen,M_ and S_gen,p_ augment while S_gen,th_ drops with the increasing values of Ra.
3. The irreversibility due thermal energy flow and applied magnetic field (S_gen,th_, S_gen,M_) augment while due to frictional forces and medium permeability (S_gen,f_, S_gen,p_) drop with augmenting magnetic field strength.
4. The augmenting values of Da enhance (S_gen,f_, S_gen,p_) while decline (S_gen,th_, S_gen,M_), respectively.
5. The Bejan number decreases with the higher buoyancy forces (higher value of Ra) whereas the Nu_ave_ enhances with the higher Ra and Da respectively.
6. The analytical expressions obtained through curve fitting show that Nu_ave_ and Be are functions of Ra, Ha and Da.
7. The agreement with the published results confirms the accuracy of the employed computational technique.

## List of symbols

$${\text{Be}}$$: Bejan number
CVFEM: Control volume based FEM
$$S_{gen}$$: Entropy generation
Da: Darcy number
Nu: Nusselt number
_*B*_: Magnetic field
*T*: Temperature
*V*: Velocity
$${\text{Ra}}$$: Rayleigh number
Ha: Hartmann number

## Greek symbols

$$\theta$$: Temperature
$\beta$: Thermal expansion coefficient
$$\sigma$$: Electrical conductivity
$$\upsilon$$: Kinetic viscosity
$$\Omega$$: Vorticity

## Subscripts

_*nf*_: Nanomaterial
_*M*_: Magnetic
_*p*_: Porous
